# Necrotizing sialometaplasia: a malignant masquerade but questionable precancerous lesion, report of four cases

**DOI:** 10.1186/s12903-020-01189-1

**Published:** 2020-07-14

**Authors:** Sun Ah. Shin, Hee Young Na, Ji Young Choe, Seung-No Hong, Ho Lee, Sunwon Park, Ji Eun Kim

**Affiliations:** 1 Department of pathology, Seoul National University Hospital, Seoul, South Korea; 2 Department of pathology, Seoul National University Bundang Hospital, Seongnam, South Korea; 3 Department of pathology, Hallym University Sacred Heart Hospital, Anyang, South Korea; 4 Department of otorhinolaryngology, Seoul National University Boramae Hospital, Seoul, South Korea; 5 Department of oral and maxillofacial surgery, Section of Dentistry, Seoul National University Boramae Hospital, Seoul, South Korea; 6 Department of radiology, Seoul National University Boramae Hospital, Dongjak-gu, Seoul, 07061 South Korea; 7 Department of pathology, Seoul National University Boramae Hospital, 20 Boramae-ro 5-gil, Dongjak-gu, Seoul, 07061 South Korea

**Keywords:** Necrotizing sialometaplasia, Differential diagnosis, Minor salivary glands

## Abstract

**Background:**

Necrotizing sialometaplasia (NSM) is an extremely rare benign lesion with an uncertain pathogenesis. The differential diagnosis of this lesion is challenging due to little familiarity with this entity and histologic similarity with carcinomas, especially mucoepidermoid carcinoma (MEC). The purpose of this study is to raise awareness about NSM, which is often overlooked or misdiagnosed as malignancy in a small biopsy.

**Methods:**

We reviewed all biopsy materials taken from the oral cavity in a single institution in Korea from 2012 to 2018 and found 4 cases of NSM out of 726. Clinicopathologic characteristics and comparison with other lesions were discussed.

**Results:**

Unlike previous reports, patients in our series were relatively young, and NSM was not related to smoking and not associated with malignancies, although one patient was misdiagnosed with MEC on the basis of the initial biopsy. High-grade squamous dysplasia was observed in one patient; however, all four patients showed excellent prognoses without further management.

**Conclusions:**

A conservative approach is recommendable for necrotizing lesions of the palate in young adults to avoid unnecessary treatment. However, careful monitoring is also required due to uncertainty of premalignant potential.

## Background

Necrotizing sialometaplasia (NSM) is a reactive, self-limiting salivary gland lesion, first described by Abram and colleagues in 1973 [1]. It is known to involve minor salivary gland tissue of the hard palate. However, other mucous glandular tissues, such as the trachea, nasal cavity, and floor of the mouth, have been reported to be involved [2–5]. In the hard palate, NSM usually appears as an ulcerative mass or swelling accompanied by pain and discomfort [6]. The incidence of NSM has been reported to account for 0.03% of all oral biopsies, but that may be underestimated because of the low recognition on this entity [7]. Histological findings are relatively characteristic, showing extensive coagulative necrosis with preserved lobular architectures and squamous metaplasia of the ductal epithelia [1]. The greatest concern regarding NSM is the risk of misinterpretation as other lesions. The most worrisome histologic mimics are mucoepidermoid carcinoma (MEC) and squamous cell carcinoma. We retrospectively investigated all oral biopsy data in a single institute to determine the real incidence of NSM and its clinicopathologic characteristics. The aim of our study is to raise awareness of this rare disease, which may be overlooked or misdiagnosed as malignancy in a small biopsy.

## Methods

All oral cavity and hard palate biopsies performed at Seoul National University Boramae Hospital, Seoul, Korea, from 2012 to 2018 were reviewed by two board-certified pathologists (S.A. Shin and J.E. Kim). Immunohistochemistry (IHC) for cytokeratin7, S100, P63, P53 and Ki-67 was performed in some selective cases using an automated immunostainer (Ventana BenchMark XT, Tuscon, AZ) with a standard protocol according to the manufacturer’s recommendation. Clinical findings, radiologic features, surgical procedures and follow-up data were retrieved from electronic medical records.

## Results

### Clinical summary

**Fig. 1:**
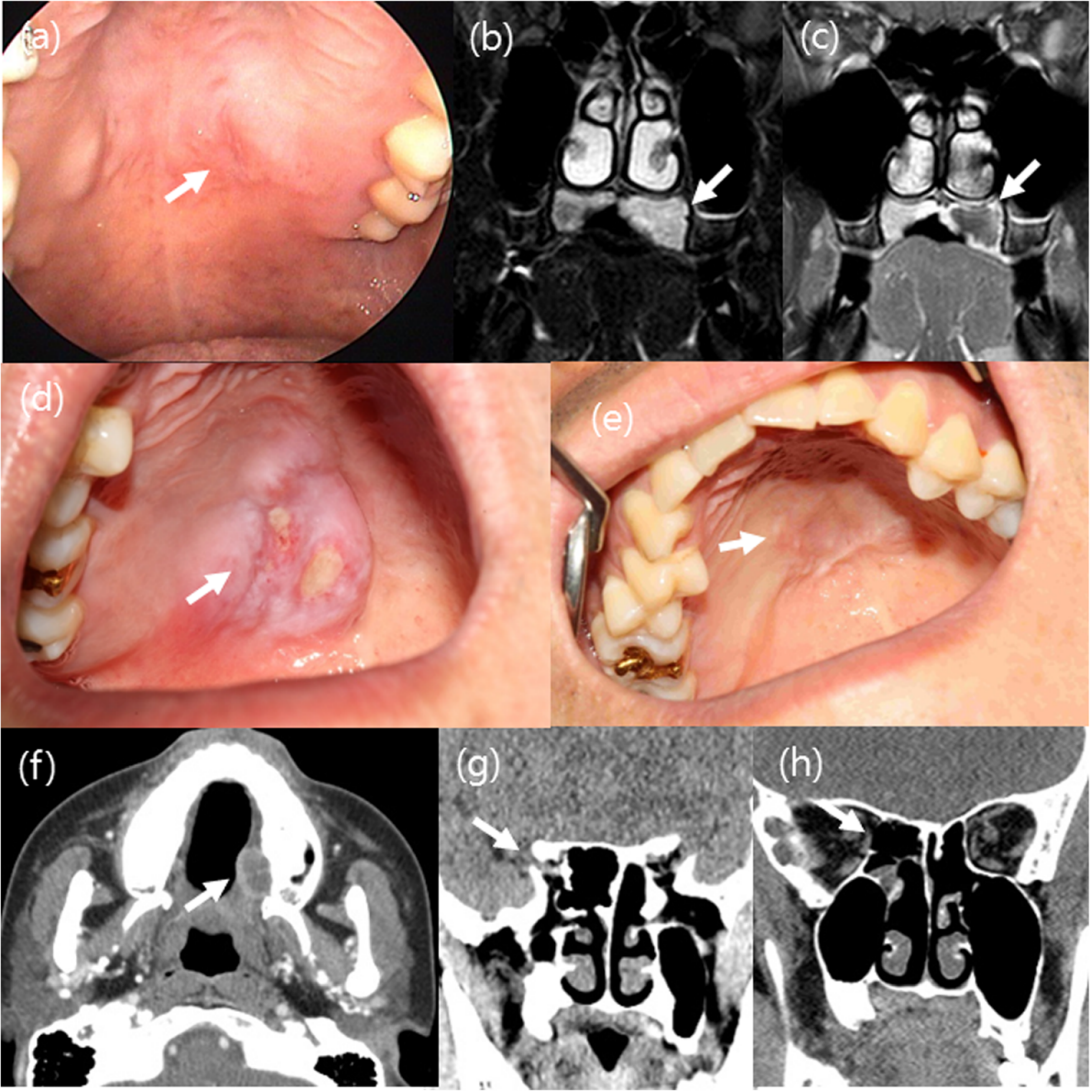
Clinicoradiologic findings of necrotizing sialometaplasia. In case 1, a bulging mass (**a**) in the left hard palate showing well defined high signal intentisity in T2 weighted coronal MRI (**b**) and peripheral enhancement and necrosis on post contrast T1-weighted coronal image (**c**). Case 3 showed an ulcerative mass (**d**) subsequently healed after 3 months (**e**). Case 2 also presented with a well demarcated peripheral enhancing mass in left hard palate by post contrast axial CT (**f**). Case 4 showed right sphenoid sinus wall defect without delineable mass in the nasal cavity or the palate (**g**). Two months after surgery, case 4 patient’s CT scan showed the same right sphenoid sinus wall defect (**h**)

Four cases of NSM out of 726 oral cavity and palate biopsy specimens were identified, accounting for 0.06%. The patients’ clinical profiles and radiologic findings are summarized in Table 1. The patients’ ages ranged from 20s to 30s. The most common presenting symptoms were pain and discomfort for several weeks. A history of trauma was noted in two patients; one had been wearing an orthodontic brace for several months, and the other had an operation on the paranasal sinuses due to prolonged cerebrospinal fluid (CSF) rhinorrhea of uncertain etiology. On radiologic examination, mass lesions were found in two patients who underwent excision of the hard palate masses (Fig. 1). Among them, one patient had been misdiagnosed with MEC on the basis of a punch biopsy and subsequently underwent radical surgery. Two other patients were diagnosed with NSM on the basis of a punch biopsy, and no additional treatment was administered. All patients were in good conditions without recurrence during follow-up periods (ranging 14–50 months).

**Table 1:** Clinical summary of 4 cases of necrotizing sialometaplasia (NSM)

	Case 1^a^	Case 2	Case 3	Case 4
Symptoms	Pain, discomfort	Mass sensation	Absent	Fever
Location	Hard palate	Hard palate	Hard palate	Hard palate
Predidsposingfactors	Orthodentic denture	Absent	Absent	Surgery (2WA)
Smoking	Denied	Denied	Denied	Denied
Radiology	T2 high intensity mass in MRI	1.7 cm low density mass (CT)	Not done	Sphenoid sinus defect, no mass
Clinical impression	Malignancy	Odontogenic abscess	Benign lesion	Inflammation
Operation	Wide resection	Resection	Biopsy	Biopsy
Pathology	NSM	NSM	NSM	NSM with dysplasia
Follow-up	NED (36 mos)	NED (18mos)	NED (50 mos)	NED (14 mos)

*WA* weeks ago, *NED* no evidence of disease, *mos* months

^a^ diagnosed with mucoepidermoid carcinoma in a punch biopsy

### Pathologic findings

**Fig. 2:**
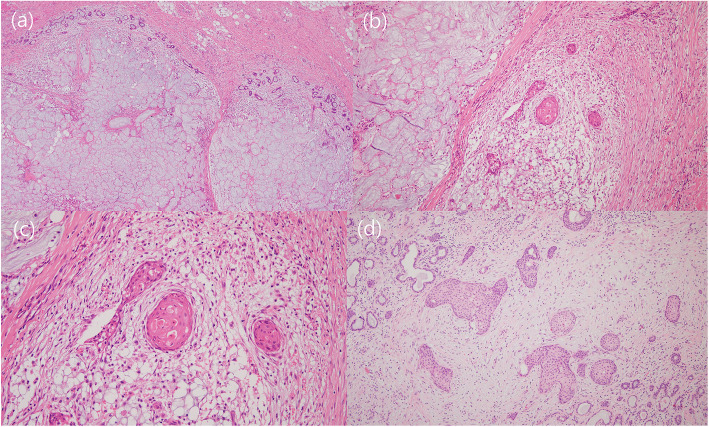
Representative microscopic features of necrotizing sialometaplasia in case 1. Extensive necrosis (**a**, × 40) with intact lobular architecture and squamous metaplasia (**b**, × 100). Metaplastic squamous cells without dysplasia are found in the inflammatory background (**c**, × 200). However, haphazardly arranged squamous cells and mucous glands causes confusion with mucoepidermoid carcinoma (**d**, × 100). (Hematoxylin Eosin)

**Fig. 3:**
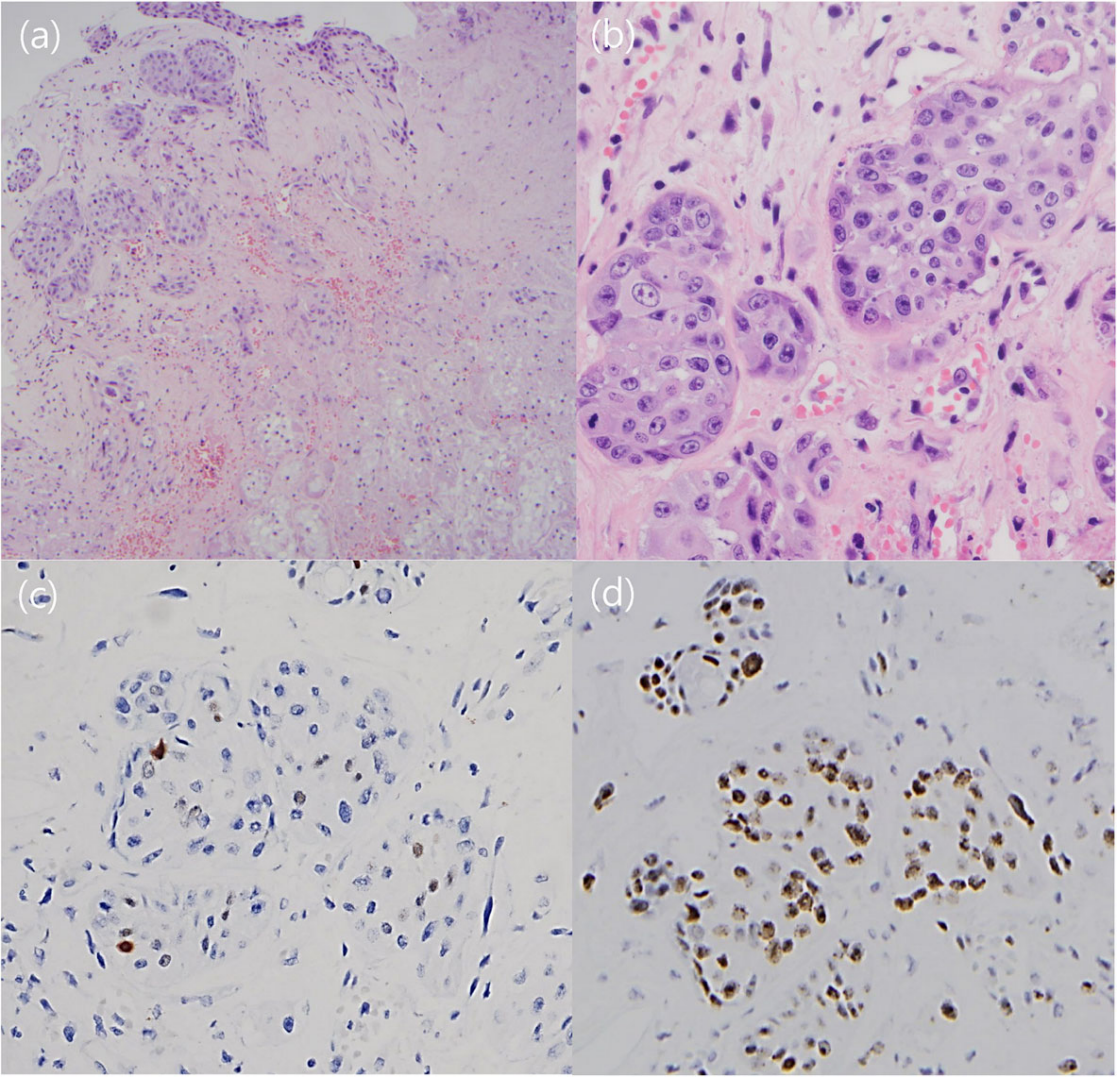
Necrotizing sialometaplasia associated with high grade dysplasia in case 4 (**a**, × 40). Marked nuclear plemorphism is evident in squamoid cells (**b**, × 200). These cells showed immunoreactivity to P53 (**c**) and high Ki-67 (**d**)

All four cases of NSM showed confluent coagulative necrosis of the mucous glands and squamous metaplasia of the ductal epithelia, which are characteristic features of NSM (Fig. 2). Cytologic atypia was not found, except in one case in which was associated with sinus wall defects and CSF rhinorrhea. That particular case showed high-grade dysplasia of the metaplastic ductal epithelial cells and overlying mucosa, a high Ki-67 labeling index reaching approximately 70% and increased P53 immunoreactivity (Fig. 3).

## Discussion

**Fig. 4:**
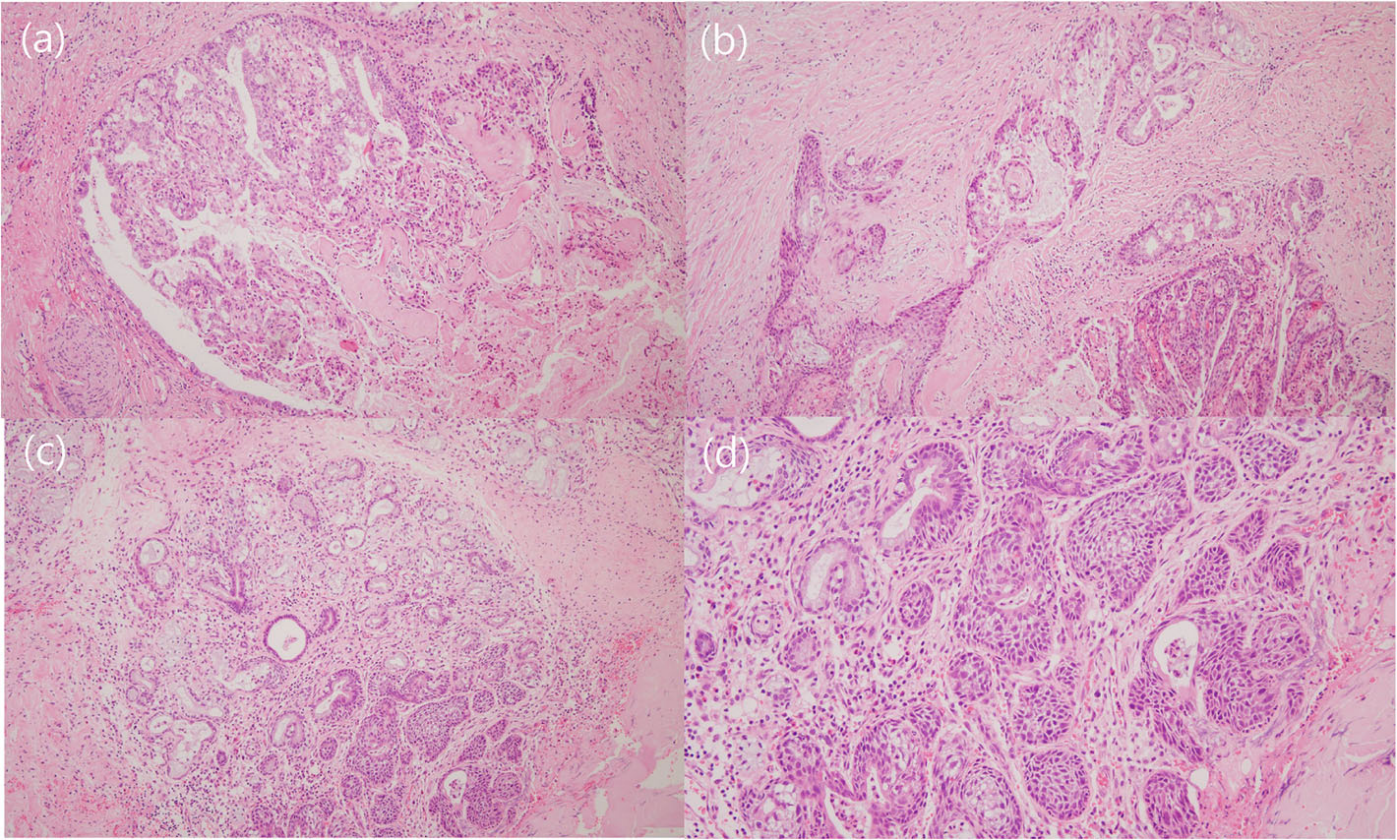
Histologic features of a case of mucoepidermoid carcinoma (MEC). Mixed infiltration of glandular cells and epidermoid cells are characteristic of MEC (**a**, × 40; **b**, × 100). However, areas mimicking necrotizing sialometaplasia are present at the periphery (**c**, × 40; **d**, × 100)

In this study, we presented 4 cases of NSM after a meticulous search of 726 oral biopsies in a single institution. Although the histologic features were almost identical, except for the presence of dysplasia in one case, the clinicoradiologic findings revealed considerable variations between ours and previously reported cases. According to the literature, NSM can occur in all ages but occurs predominantly after middle age, with a higher incidence in males than in females [6]. However, patients in our series were much younger than middle-aged and showed no sex differences. Mass lesions were present in half of our cases. Conventionally, NSM has been reported to present as a circumscribed ulcer with a diameter of 1 to 3 cm [8]. We suggest that the primary reason for these discrepancies is the difference in the case selection. Many of the previous studies included focal or secondary NSM of the background mucous glands in mass-forming salivary lesions. In this study, we included cases in which NSM was the main pathology, excluding any cases associated with malignancies. As described in the literature, NSM-like morphologic changes can be seen in the periphery of the cancer-involved salivary glands, which raises suspicion that NSM might be a precancerous lesion [9]. We also discovered some cases of salivary gland carcinoma accompanied by squamous metaplasia of the surrounding ductal epithelia (Fig. 4). However, a typical confluent infarct with intact lobular architecture was lacking in those lesions.The etiology of NSM is still uncertain. Naturally, ischemia of the salivary gland lobule resulting from vascular injury and physical or chemical trauma have been suggested to be related to the pathogenesis of NSM [2, 10]. Atherosclerotic changes were proposed as etiologies when the disease was first described [1, 8], and following studies suggested that previous surgeries, dentures, smoking and upper respiratory infection were predisposing factors [6]. An association with local anesthetic injection has also been demonstrated in animal experiments [11]. Recently, some studies have reported cases of NSM associated with eating disorders in young females. Some patients reported a history of consuming ice chips, directly affecting vasoconstriction, while others presented regurgitation of gastric acid due to recurrent vomiting in anorexia nervosa or gastroesophageal reflux disease [12–16]. In our series, only two patients had evident predisposing factors: wearing a post-orthodontic retainer in case 1 and long-standing inflammation and recent surgery in case 4. The etiology of NSM was unclear in the other two cases because the likelihood of vascular insufficiency is low in young, healthy individuals. In case 4, NSM might have been caused by either recent surgery or longstanding chronic inflammation, as reported in previous studies [6]. However, obvious dysplastic epithelial changes suggest possible premalignant potential and can mimic squamous cell carcinoma.

The main differential diagnosis of NSM include MEC, squamous cell carcinoma, subacute necrotizing sialadenitis (SANS), and mucocele. Abrams and colleagues presented five morphologic criteria of NSM: 1) massive infarction, 2) bland nuclear features, 3) simultaneous metaplasia of the ducts and acini, 4) prominence of inflammatory granulation tissue, and 5) maintenance of the lobular structures [1]. In general, the absence of nuclear pleomorphism or atypism is the most authentic parameter supporting a benign nature, but these features are not applicable in the exclusion of MEC. Low-grade MEC can present relatively bland cytologic features in either epidermoid cells or glandular cells. Some researchers suggested that IHC using a panel of P53, P63 and Ki-67 could be helpful for the differentiation of NSM and MEC [8]. However, ductal epithelial cells in case 4 showed marked atypia corresponding to high grade dysplasia and high Ki-67 and P53. Application of IHC seems to have limited diagnostic value in a differential diagnosis of MEC, because MEC itself lacks any specific or pathognomonic protein biomarkers. Morphologic patterns, such as stromal invasion and disruption of lobular architectures, are more reliable supporting factors in MEC than in NSM. However, these features are not easily recognizable in small biopsy specimens. According to Brannon et al., NSM was misdiagnosed as malignancy in 21% of the preoperative biopsied cases they collected [6]. Although MEC and squamous cell carcinoma are the most common types of misdiagnosed malignancies on the basis of biopsy, NSM has also been misdiagnosed as acinic cell carcinoma, verrucous carcinoma, and ductal carcinoma. The incidence of pure squamous cell carcinoma in the salivary glands is extremely low; this should be kept in mind when squamous cell lesions are encountered in this area [17]. Among the benign entities, SANS and mucocele can be considered in differential diagnoses. SANS is also a benign disease that usually occurs in the minor salivary gland of the hard palate. The principal histopathology of SANS is inflammation without squamous metaplasia. Necrosis is rare in SANS, and if present, it is visible only focally [18].

Although the clinical course of NSM is self-limiting, the premalignant potential of NSM has long been pointed out. Association with other malignancies, such as epithelial-myoepithelial carcinoma or adenoid cystic carcinoma, and even malignant lymphoma have been reported to coexist with NSM [9, 19–22]. There are still controversial issues regarding the application of strict criteria for NSM in these lesions due to the possibility of overdiagnosing NSM, as shown in some previous reports. Nevertheless, careful evaluation and follow-up is necessary for NSM patients. It should be noted that delayed self-healing may require re-evaluation or resection of the lesion for a correct diagnosis and vice versa.

## Conclusion

NSM is a disease that can mimic malignancy, especially in small biopsy specimens. It is important to be aware of this rare disease and understand its clinicopathologic findings to avoid unnecessary intervention. The premalignant potential of NSM should be detemined in future large-cohort studies.

## Data Availability

Not applicable. The clinicoradiologic findings of all four cases were presented in detail (Table 1).

## Abbreviations

NSM: Necrotizing sialometaplasia
MEC: Mucoepidermoid carcinoma
IHC: Immunohistochemistry
CSF: Cerebrospinal fluid
SANS: Subacute necrotizing sialadenitis

## References

[1] Abrams AM, Melrose RJ, Howell FV (1973). Necrotizing sialometaplasia. A disease simulating malignancy. Cancer..

[2] Batsakis JG, Manning JT (1987). Necrotizing sialometaplasia of major salivary glands. J Laryngol Otol.

[3] Devine M, Sammut S, Conn B, Lopes V (2014). Necrotising sialometaplasia in the floor of mouth. Oral Maxillofac Surg.

[4] Maisel RH, Johnston WH, Anderson HA, Cantrell RW (1977). Necrotizing sialometaplasia involving the nasal cavity. Laryngoscope..

[5] Romagosa V, Bella MR, Truchero C, Moya J (1992). Necrotizing sialometaplasia (adenometaplasia) of the trachea. Histopathology..

[6] Brannon RB, Fowler CB, Hartman KS (1991). Necrotizing sialometaplasia. A clinicopathologic study of sixty-nine cases and review of the literature. Oral Surgery, Oral Med Oral Pathol.

[7] Mesa ML, Gertler RS, Schneider LC (1984). Necrotizing sialometaplasia: frequency of histologic misdiagnosis. Oral Surgery, Oral Med Oral Pathol..

[8] Carlson DL (2009). Necrotizing sialometaplasia a practical approach to the diagnosis. Arch Pathol Lab Med.

[9] Zhurakivska K, Maiorano E, Nocini R, Mignogna MD, Favia G, Troiano G (2019). Necrotizing sialometaplasia can hide the presence of salivary gland tumors: a case series. Oral Dis.

[10] Arguelles MT, Viloria JB, Talens MC, McCrory TP (1976). Necrotizing sialometaplasia. Oral Surgery, Oral Med Oral Pathol..

[11] Shigematsu H, Shigematsu Y, Noguchi Y, Fujita K (1996). Experimental study on necrotizing sialometaplasia of the palate in rats: role of local anesthetic injections. Int J Oral Maxillofac Surg.

[12] Gilowski Ł, Wiench R, Polakiewicz-Gilowska A, Dwornicka K (2014). Necrotizing sialometaplasia of the palatal mucosa in patient with history of anorexia: review and case report. Am J Otolaryngol - Head Neck Med Surg.

[13] Janner SFM, Suter VGA, Reichart PA, Altermatt HJ, Bornstein MM (2014). Bilateral necrotizing sialometaplasia of the hard palate in a patient with bulimia: a case report and review of the literature. Quintessence Int (Berl).

[14] Schöning H, Emshoff R, Kreczy A (1998). Necrotizing sialometaplasia in two patients with bulimia and chronic vomiting. Int J Oral Maxillofac Surg.

[15] Scully C, Eveson J (2004). Sialosis and necrotising sialometaplasia in bulimia; a case report. Int J Oral Maxillofac Surg.

[16] Solomon LW, Merzianu M, Sullivan M, Rigual NR (2007). Necrotizing sialometaplasia associated with bulimia: case report and literature review. Oral Surgery, Oral Med Oral Pathol Oral Radiol Endodontology.

[17] Farthing PM, Speight PM (2006). Problems and pitfalls in oral mucosal pathology. Curr Diagn Pathol.

[18] Fowler CB, Brannon RB (2000). Subacute necrotizing sialadenitis: report of 7 cases and a review of the literature. Oral Surg Oral Med Oral Pathol Oral Radiol Endod.

[19] Dominguez-Malagon H, Mosqueda-Taylor A, Cano-Valdez AM (2009). Necrotizing sialometaplasia of the palate associated with angiocentric T-cell lymphoma. Ann Diagn Pathol.

[20] Lee DJ, Ahn HK, Koh ES, Rho YS, Chu HR (2009). Necrotizing Sialometaplasia accompanied by adenoid cystic carcinoma on the soft palate. Clin Exp Otorhinolaryngol.

[21] Poulson TC, Greer RO, Ryser RW (1986). Necrotizing sialometaplasia obscuring an underlying malignancy: report of a case. J Oral Maxillofac Surg.

[22] Yoshioka T, Harada M, Umekita Y, Taguchi S, Higashi M, Nakamura D (2010). Necrotizing sialometaplasia of the parotid gland associated with angiocentric T-cell lymphoma: a case report and review of the literature: case report. Pathol Int.

